# Placental Site Trophoblastic Tumor: Two Rare Case Report and Literature Review Within a Decade

**DOI:** 10.1155/crog/9928611

**Published:** 2026-04-10

**Authors:** Yiqi Guan, Jinsong Han, Kun Zhang, Yiting Wang

**Affiliations:** ^1^ Department of Obstetrics and Gynecology, Peking University Third Hospital, National Clinical Research Center for Obstetrics and Gynecology, Beijing, 100191, China, puh3.net.cn

**Keywords:** case report, diagnosis, placental site trophoblastic tumor, treatment

## Abstract

This case report presents two rare cases of placental site trophoblastic tumor (PSTT) with detailed clinical presentations, diagnostic challenges, and long‐term treatment outcomes. PSTT is a rare form of gestational trophoblastic neoplasia with highly heterogeneous clinical manifestations, making it a valuable addition to the medical literature.

## 1. Introduction

Placental site trophoblastic tumor (PSTT) is an exceptionally rare form of gestational trophoblastic neoplasia characterized by highly heterogeneous clinical manifestations, posing significant diagnostic and therapeutic challenges. Due to its rarity, with only ~300 cases reported worldwide and nonspecific symptoms, PSTT is frequently misdiagnosed. This report presents two rare cases managed at our institution over the past decade, detailing their distinct clinical presentations, diagnostic complexities, and successful treatment outcomes, contributing valuable insights to the limited body of literature on this uncommon malignancy.

## 2. Case Report

### 2.1. Case 1

A 31‐year‐old female, gravida 5, para 2, had three induced abortions in early pregnancy prior to 2010 and underwent one cesarean section in 2011. On April 1, 2014, she had a full‐term cesarean section at local hospital due to a scarred uterus. Postoperatively, she experienced hemorrhagic lochia, which gradually decreased. Starting on May 1, 2014, she had unexpected vaginal bleeding that was heavier than menstruation, without abdominal pain. On May 6, she visited a local hospital where her hemoglobin was 70 g/L and her serum human chorionic gonadotropin (HCG) was 3404 mIU/mL. Ultrasound revealed a heterogeneous mass in the posterior wall of the uterus measuring 3.8 cm × 3.5 cm, raising suspicion for placental implantation. After receiving a blood transfusion to correct anemia, she was prescribed oral mifepristone 25 mg bid for 7 days. A dilation and curettage (D&C) was performed on May 13, with pathology indicating a large amount of clot with suspicious chorionic shadowing. On May 14, her serum HCG rose to 8087 mIU/mL, and ultrasound showed a solid mass in the uterus measuring 5.8 cm × 4.5 cm × 6.1 cm, with a high suspicion for choriocarcinoma. On May 17, she presented to our hospital’s outpatient with a small amount of vaginal bleeding; her uterus was enlarged as if at 7 weeks of gestation, and her serum HCG was 14,093 mIU/mL. Ultrasound indicated a mixed echogenicity mass in the uterine cavity measuring 5.8 cm × 4.9 cm × 5.4 cm, with unclear demarcation from the myometrium within some blood flow signals, raising the suspicion of gestational trophoblastic tumor (GTN). She was then admitted to hospitalization on the same day.

After admission, a pulmonary CT scan showed no metastasis. Pelvic MRI with contrast revealed an abnormal echo within the uterine cavity measuring 5.3 cm × 4.7 cm × 5.6 cm, with invasion of the left and posterior walls of the uterus (Figure 1). The consultation result from our hospital’s pathology department on the curettage specimen indicated that most of the tissue was composed of blood clots, with a small amount of highly degenerated villi and decidual tissue. No trophoblastic cells or neoplastic changes were observed, and placental implantation was considered as a possible diagnosis. On May 22, a single intramuscular injection of MTX 80 mg was administered. On May 29, hysteroscopic resection of the lesion was performed. Intraoperatively, a purple‐red mass with a diameter of 6 cm was observed, which was closely related to the lower segment of the uterus and the left posterior wall. The mass was separated by an electrosurgical loop scraping along the endometrium and then removed in pieces using forceps. No obvious residual tissue was observed upon reinspection of the uterine cavity. The intraoperative blood loss was 20 mL. On May 30, the serum HCG level was rechecked at 3100 mIU/mL. The postoperative pathology revealed a large amount of necrotic and degenerative tissue with significant bleeding. Focal areas of proliferative trophoblastic cells with certain atypia were observed, and suspicious villous shadows were seen locally. Combined with the medical history, the diagnosis was inclined towards an exaggerated reaction of the placental site, while PSTT could not be completely ruled out. Immunohistochemical results: CD146 (+), cytokeratin (CK) mixed (+), hCG partially weak (+), human placental lactogen (hPL) (+), Ki‐67 (~30% +), SMA (−).

Figure 1Preoperative hysteroscopy (a) and preoperative hysterectomy (b) pelvic MRI images of Case 1.

**Figure:**
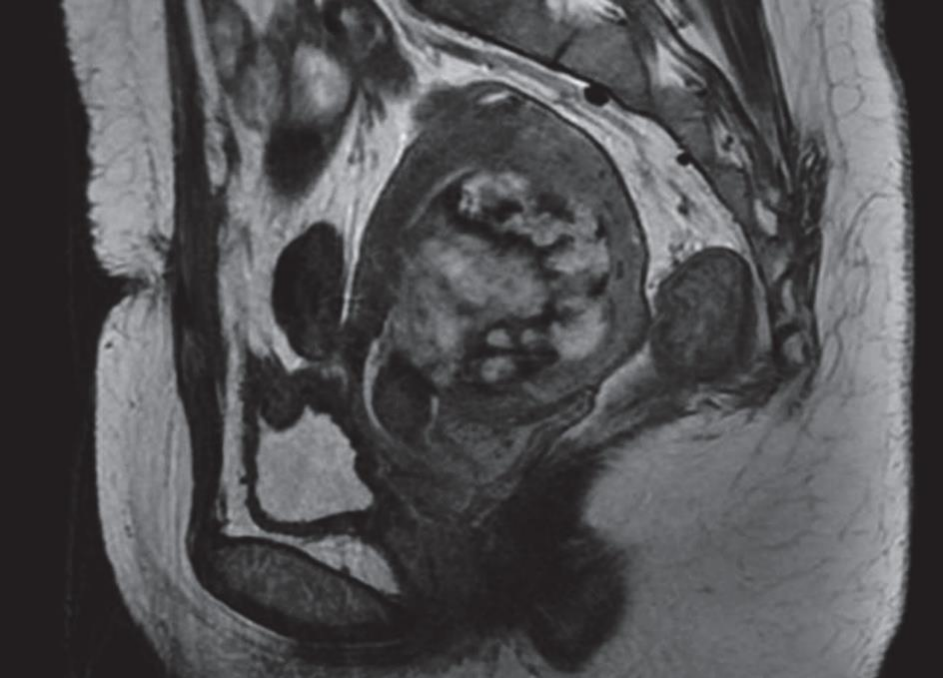
(a)

**Figure:**
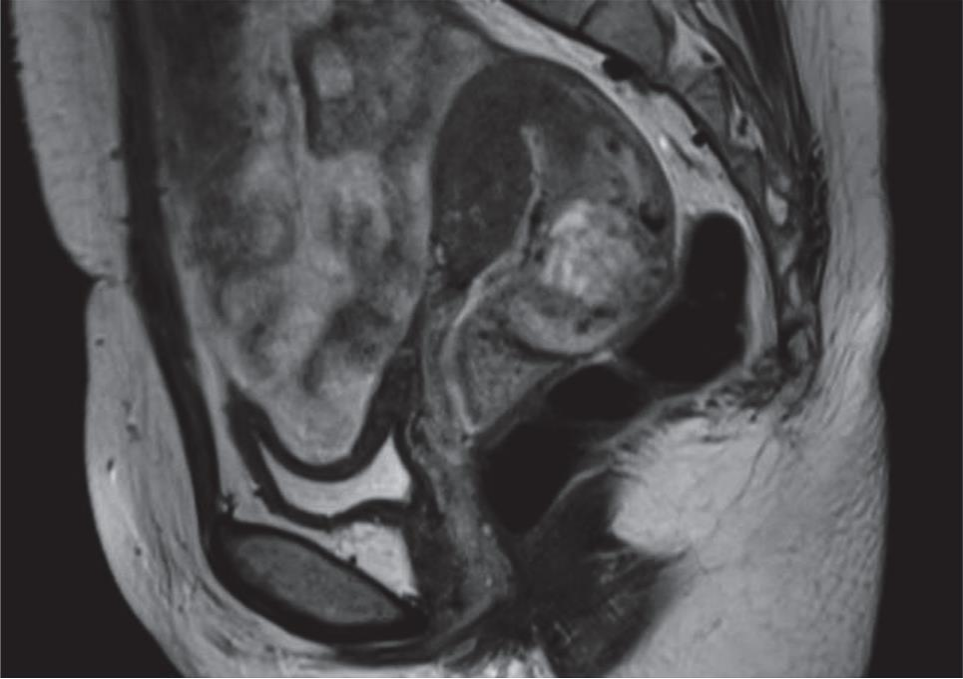
(b)

**Figure 2:**
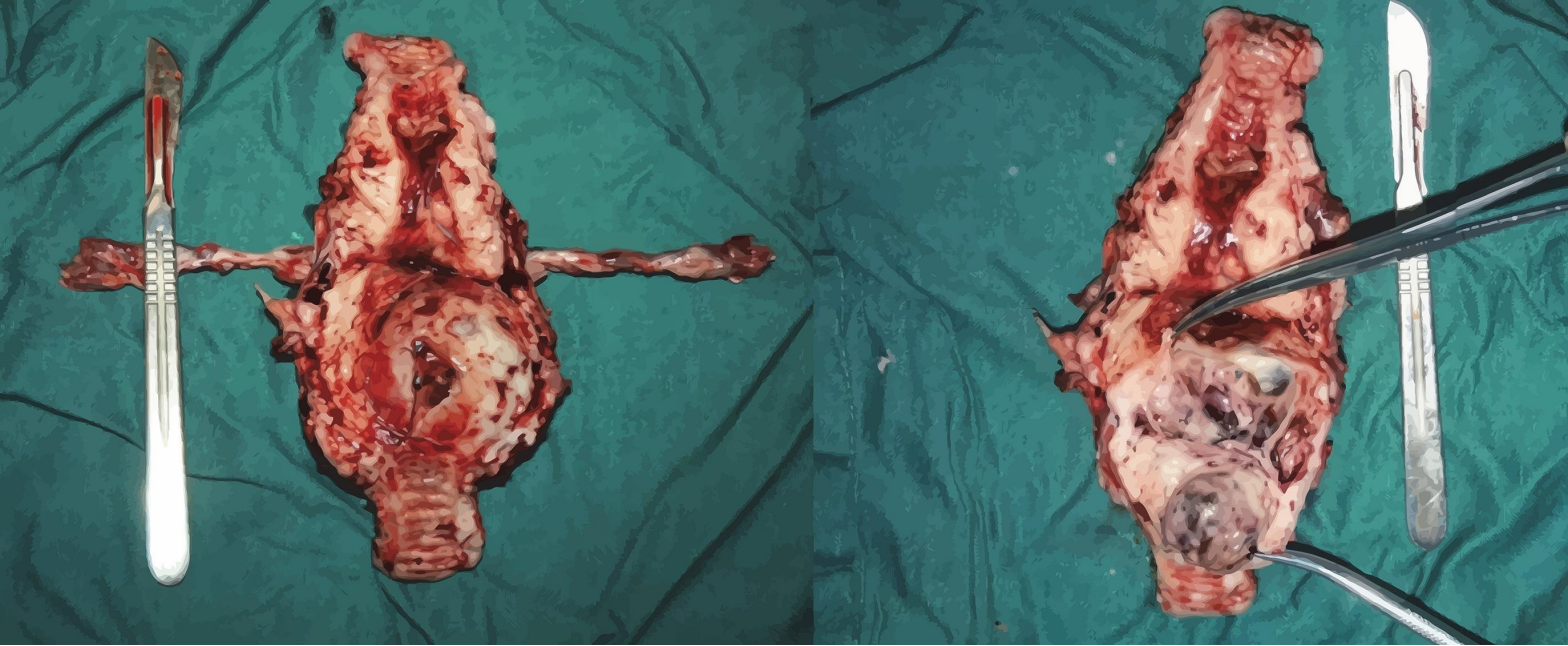
Gross specimen after total hysterectomy and bilateral salpingectomy in Case 1.

After discharge, close monitoring was conducted. On June 19, serum HCG was measured at 2266 mIU/mL. On June 24, serum HCG was rechecked at 11,551 mIU/mL. Ultrasound suggested a 31 mm × 26 mm hyperechoic area in the posterior wall of the lower segment of the uterus, with rich blood flow signals. Pelvic MRI suggested a uterine space‐occupying lesion with hemorrhage, reduced in size compared to before, ~2.5 cm × 3.8 cm × 3.4 cm, invading the left and posterior walls of the uterus. Considering the medical history, the possibility of PSTT was highly suspected. On June 26, laparoscopic total hysterectomy and bilateral salpingectomy were performed at our hospital. Upon sectioning the uterus, a purple‐blue, soft lesion with a diameter of over 2 cm was observed in the posterior wall of the lower segment, which was friable (Figure 2). Postoperative pathology: the morphology and immunohistochemistry were consistent with PSTT. The tumor measured ~3.5 cm × 2.5 cm × 2.2 cm, infiltrating the myometrium, with no definite nerve invasion or vascular tumor thrombus observed (Figure 3).

Figure 3Histopathological sections of Case 1: (a) HE staining of curettage specimen at 20× magnification; (b) Ki‐67 staining of curettage specimen; (c) HE staining of hysterectomy specimen at 20× magnification; (d) Ki‐67 staining of hysterectomy specimen.

**Figure:**
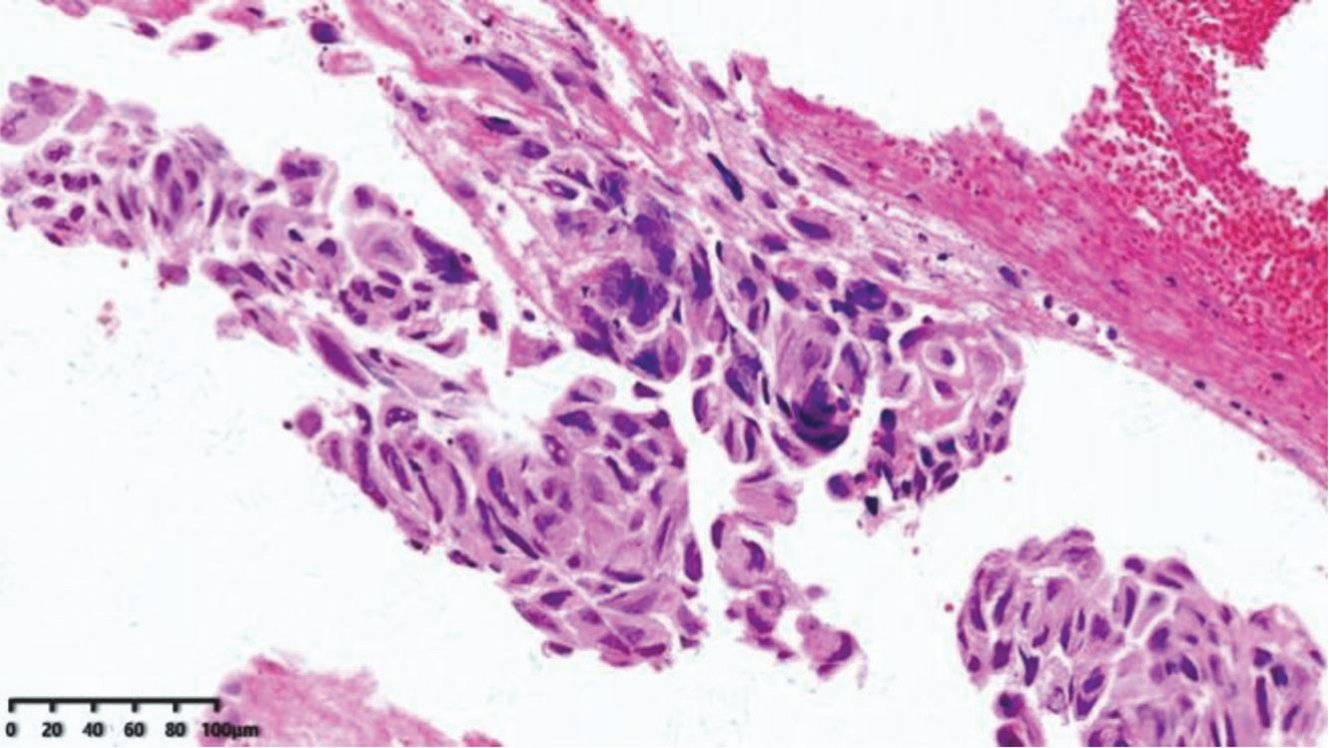
(a)

**Figure:**
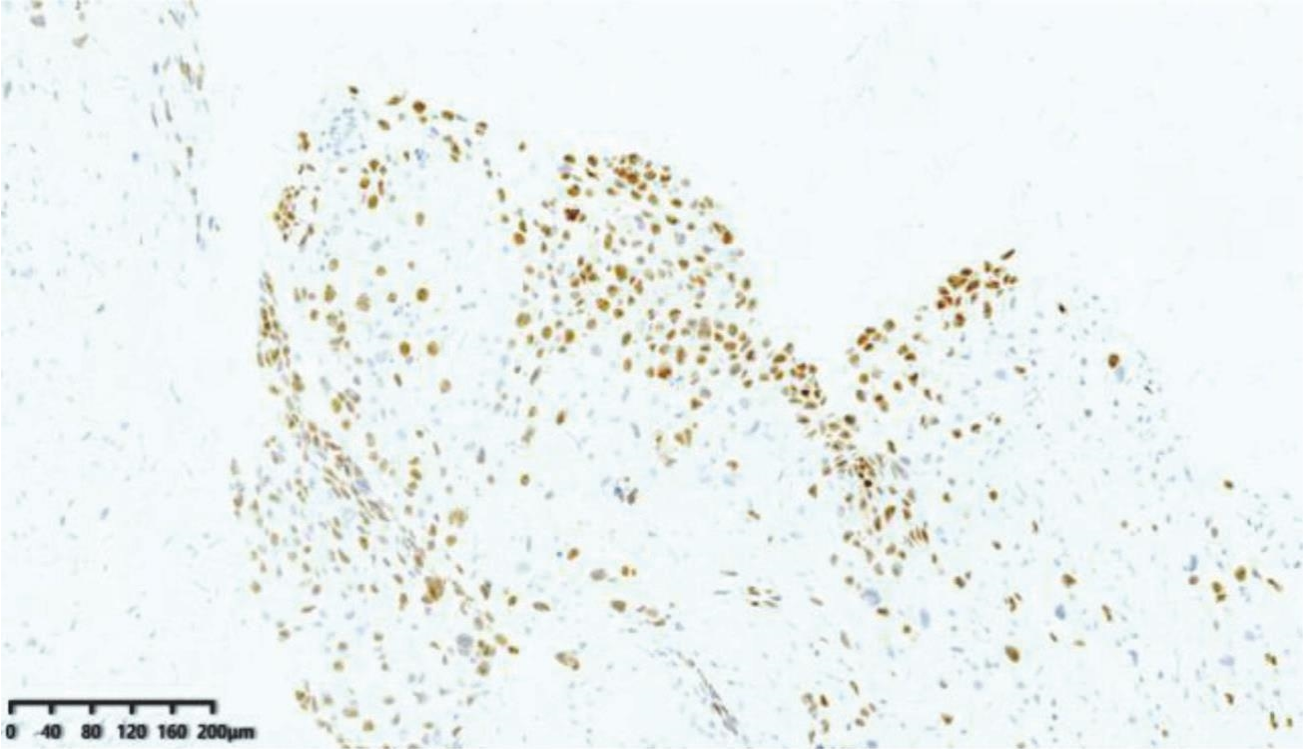
(b)

**Figure:**
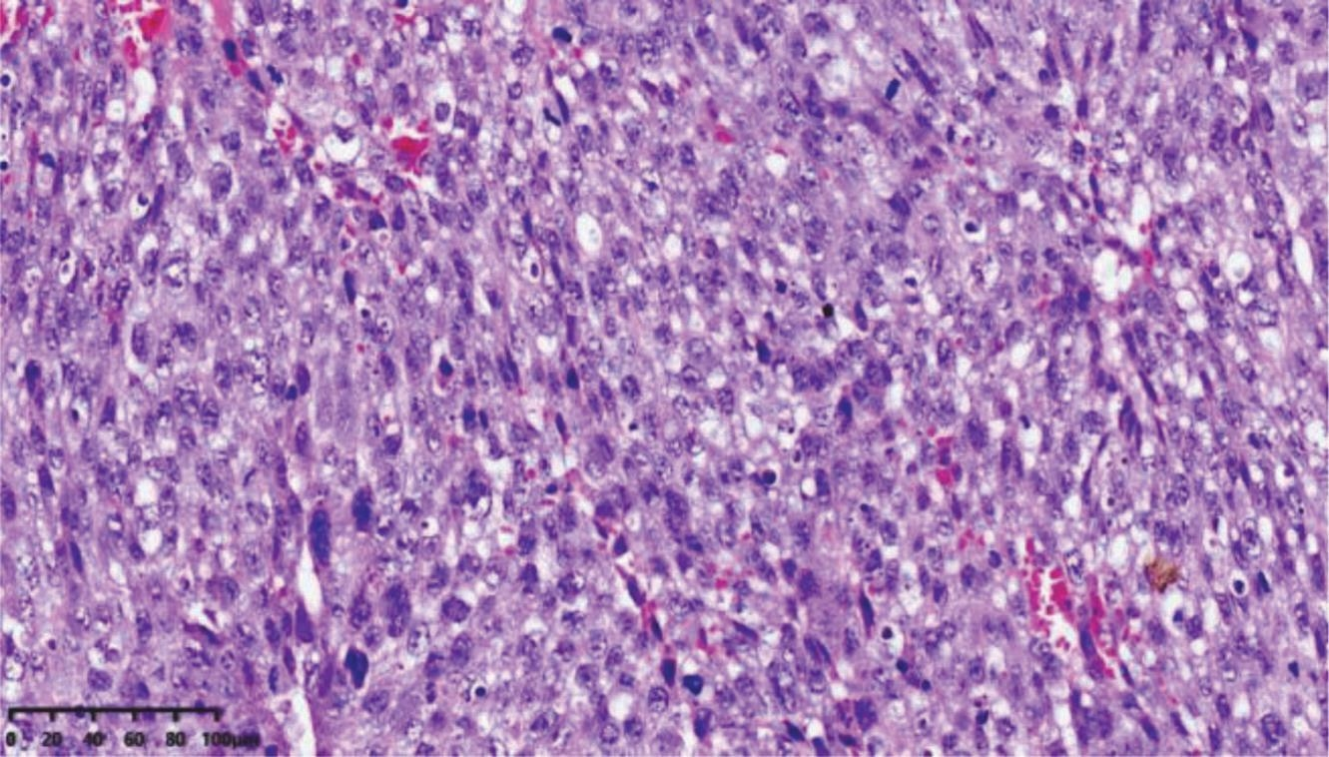
(c)

**Figure:**
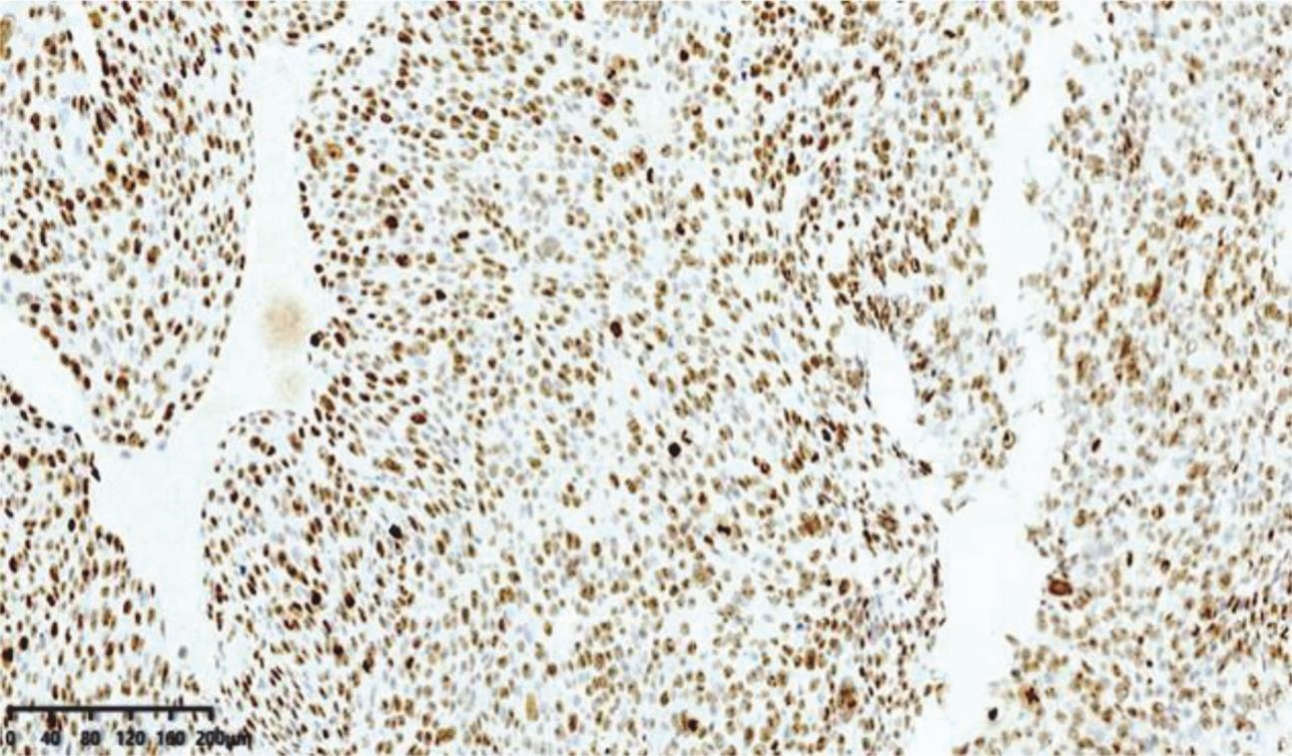
(d)

**Figure 4:**
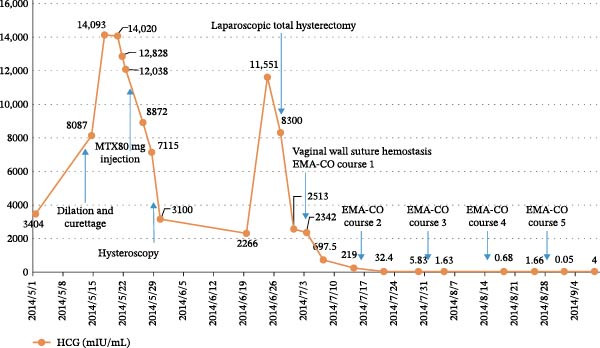
Treatment course and changes in serum HCG levels in Case 1.

On July 3 (7 days postoperatively), the patient suddenly experienced massive vaginal bleeding, totaling ~400 mL. Physical examination revealed a protruding purple‐blue nodule on the vaginal sidewall at the 2 o’clock position, with a diameter of about 2 cm and active bleeding. A vaginal wall metastasis was suspected, and a vaginal wall suture hemostasis procedure was performed, which successfully stopped the bleeding. With a diagnosis of stage II PSTT, the patient underwent EMA‐CO chemotherapy from July 3 to September 4. After the third course, serum HCG levels normalized. The patient then received two additional courses, making a total of five chemotherapy courses (Figure 4). After chemotherapy, regular monitoring of serum HCG and pulmonary CT scans was conducted for 2 years, with no significant abnormalities detected. During the 10‐year follow‐up in December 2024, no recurrence was observed.

### 2.2. Case 2

A 28‐year‐old female, gravida 1, para 1, with a history of cesarean section in 2021. The patient usually has regular menstrual cycles. Her last menstrual period was on March 9, 2024. She was scheduled for a hysteroscopy due to decreased menstrual flow. On March 17, preoperative HCG was 32.31 mIU/mL. On March 19, a recheck of serum HCG showed 23.22 mIU/mL. Local hospital considered a biochemical pregnancy and prescribed oral mifepristone 25 mg once daily for 6 days. On March 25, HCG was rechecked at 35.83 mIU/mL, and ultrasound showed endometrial fluid collection. She then took oral mifepristone 25 mg twice daily for another 6 days. Over the next 3 weeks, HCG levels fluctuated between 32 and 35 mIU/mL. On April 15, a follow‐up ultrasound showed no gestational sac in the uterine cavity. After taking traditional Chinese medicine for 10 days, there was no significant change in HCG levels or ultrasound findings.

**Figure 5:**
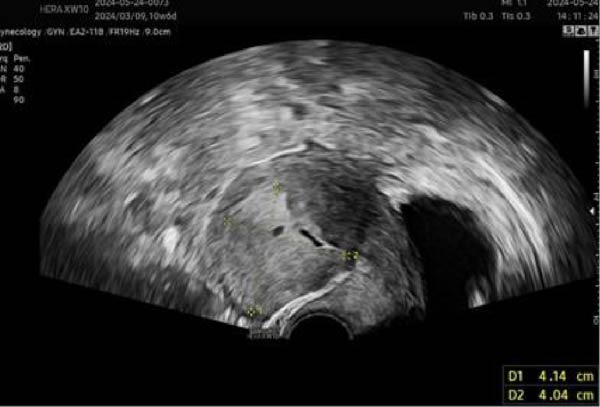
Initial gynecological ultrasound image of Case 2 on May 24, 2024, at our hospital.

On May 24, the patient visited our hospital. Her serum HCG was 37.45 mIU/mL. Ultrasound showed a 4.1 cm × 4.0 cm hypoechoic mass with unclear boundaries in the anterior myometrium, with a 0.6 cm × 0.4 cm anechoic area within and blood flow signals in the hypoechoic tissue (Figure 5). Considering the possibility of GTN, the patient was admitted to hospitalization.

After admission, a pulmonary CT scan was normal. Pelvic MRI showed a lesion within the anterior myometrium, measuring ~45 mm × 29 mm × 45 mm, with isointense T1 and slightly prolonged T2 signal. On DWI, the lesion exhibited marked high signal intensity, and the ADC signal was slightly reduced. The adjacent junctional zone was poorly visualized, and the endometrium was thinned with unclear boundaries.

**Figure 6:**
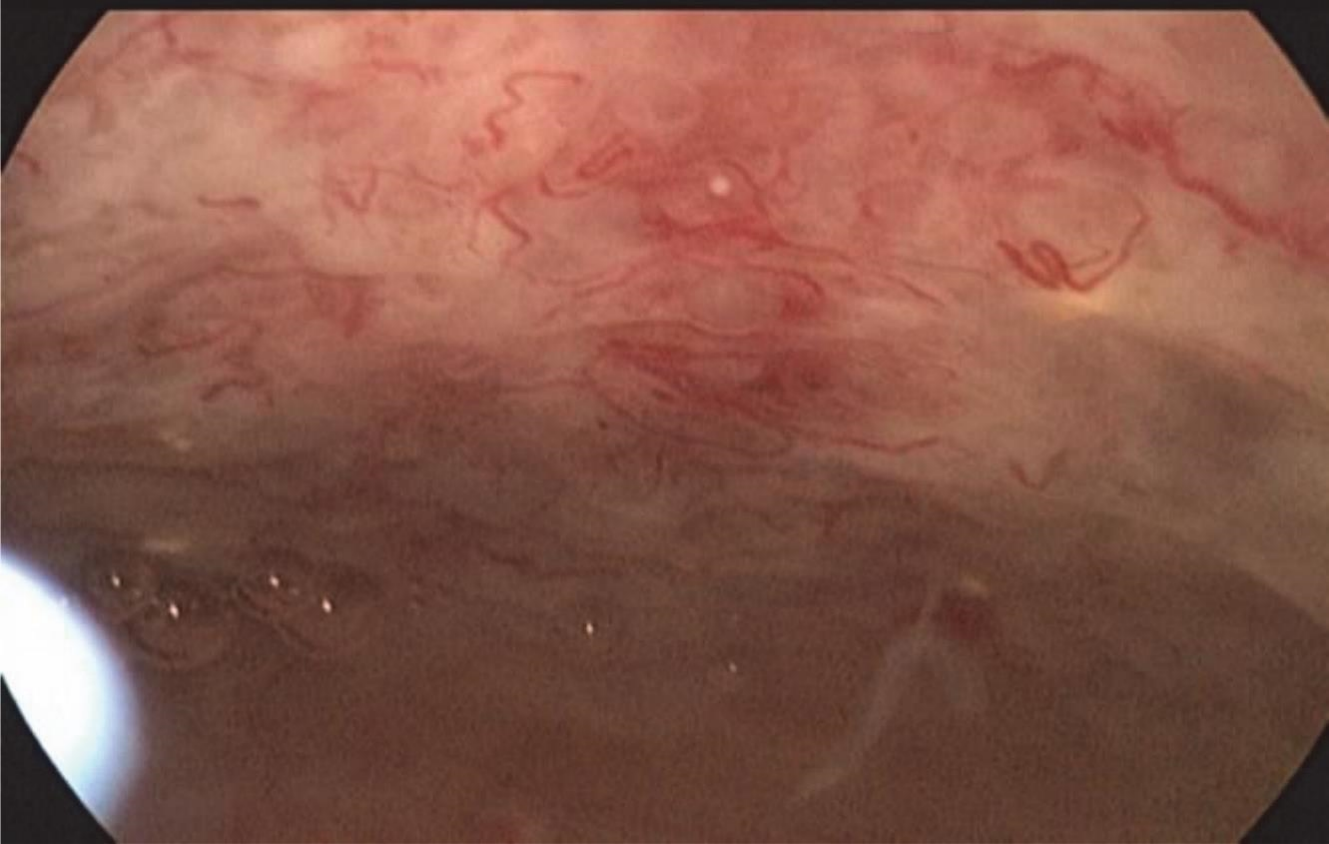
Findings from hysteroscopy in Case 2.

On May 27, hysteroscopy with endometrial biopsy was performed. Intraoperatively, the anterior endometrium appeared slightly rough, with numerous atypical blood vessels visible (Figure 6). Intraoperative frozen section pathology of the endometrial curettage revealed a small amount of endometrial and decidual tissue, with no definite evidence of neoplastic lesion. Upon incision of the anterior myometrium, normal myometrial tissue was not observed, and fibrous tissue was seen. Postoperative pathology revealed infiltrative growth of intermediate trophoblasts with relatively uniform morphology within the myometrium. Combined with the medical history, histological features, immunohistochemical, and molecular detection results, the diagnosis was consistent with PSTT. Immunohistochemical results: HCG (focal cells +), HLA‐G (+), P63 (−), SALL4 (−), CD146 (+), hPL (+), Ki‐67 and CK8/18 double staining (~5% +).

Communication with the patient was conducted. Given that PSTT is not sensitive to chemotherapy and the follow‐up pelvic MRI showed a lesion in the anterior myometrium, measuring ~45 mm × 29 mm × 45 mm with isointense T1 and slightly prolonged T2 signal, which was essentially unchanged from the previous examination (Figure 7), and considering that the lesion had invaded the full thickness of the anterior myometrium, a hysterectomy was recommended.

Figure 7Preoperative hysteroscopy (a) and preoperative hysterectomy (b) pelvic MRI images of Case 2.

**Figure:**
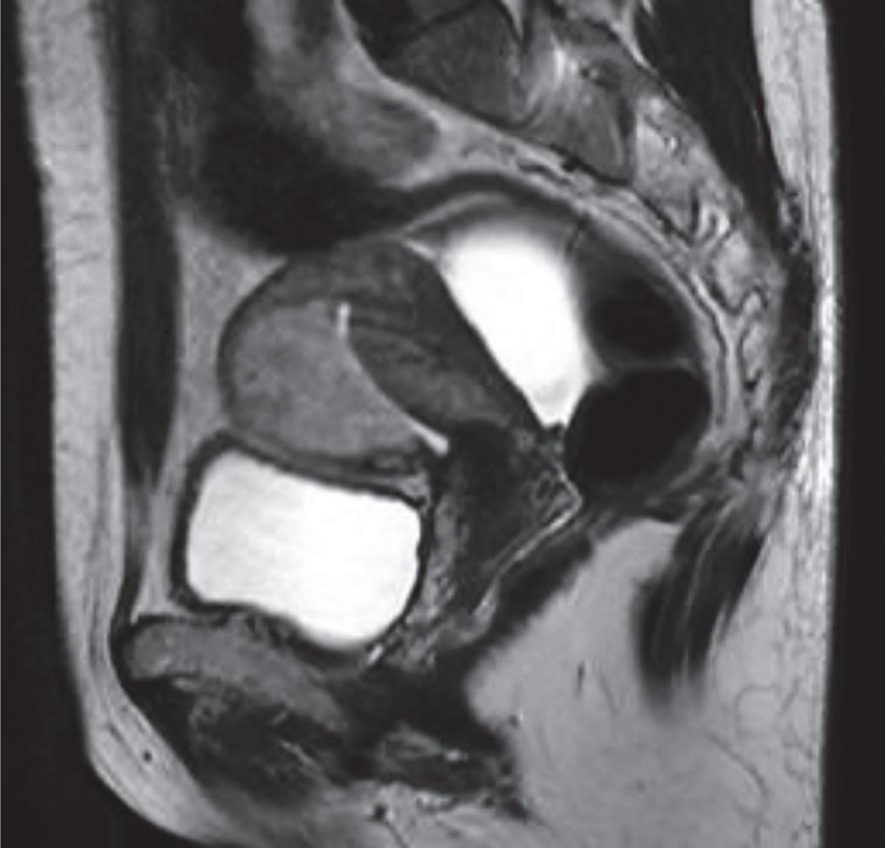
(a)

**Figure:**
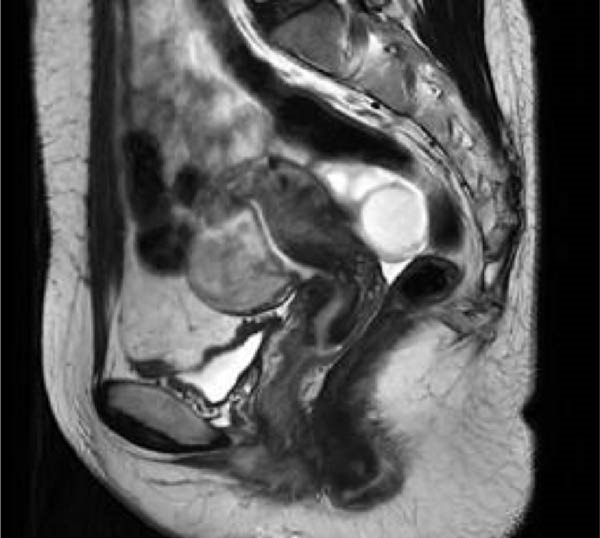
(b)

**Figure 8:**
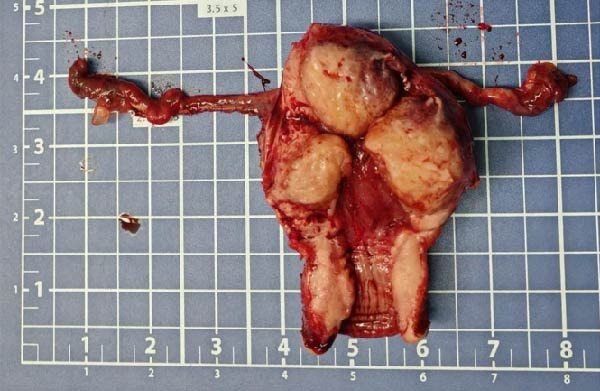
Gross specimen after total hysterectomy and bilateral salpingectomy in Case 2.

**Figure 10:**
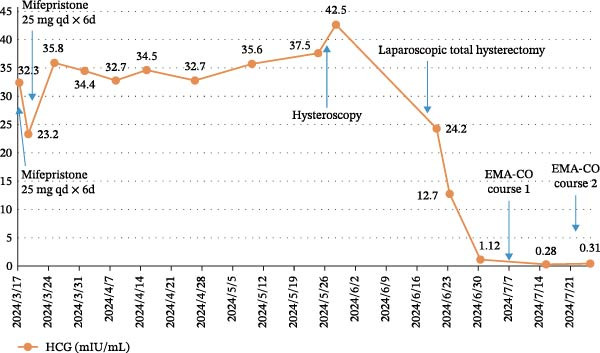
Treatment course and changes in serum HCG levels in Case 2.

On June 22, 2024, the patient underwent laparoscopic total hysterectomy, bilateral salpingectomy, and removal of ovarian cysts. Intraoperatively, the lesion was observed in the anterior myometrium, penetrating the entire layer, with a yellowish cut surface and firm consistency (Figure 8). Postoperative pathology revealed PSTT. The tumor had invaded the deep myometrium without involving the lower segment of the uterus (Figure 9). One week after the hysterectomy (June 30), the serum HCG level decreased to normal. Although the staging was I, given that the patient had high‐risk factors, she received two courses of intravenous EMA‐CO chemotherapy from July 9 to July 31, 2024. Since the completion of chemotherapy, serum HCG levels have remained within the normal range during follow‐up until May 2025 (Figure 10).

Figure 9Histopathological sections of Case 2: (a) HE staining at 20× magnification showing thick‐walled vessels replaced by trophoblastic cells; (b) dual staining for Ki‐67 and CK8/18.

**Figure:**
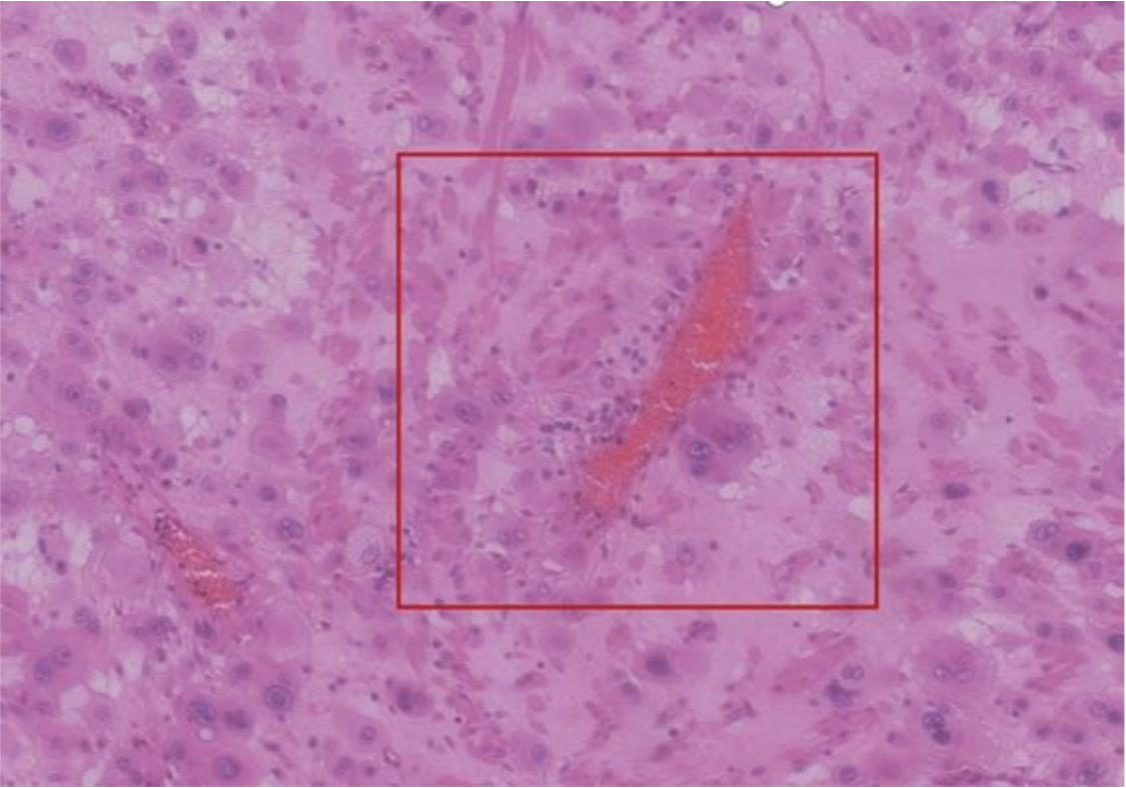
(a)

**Figure:**
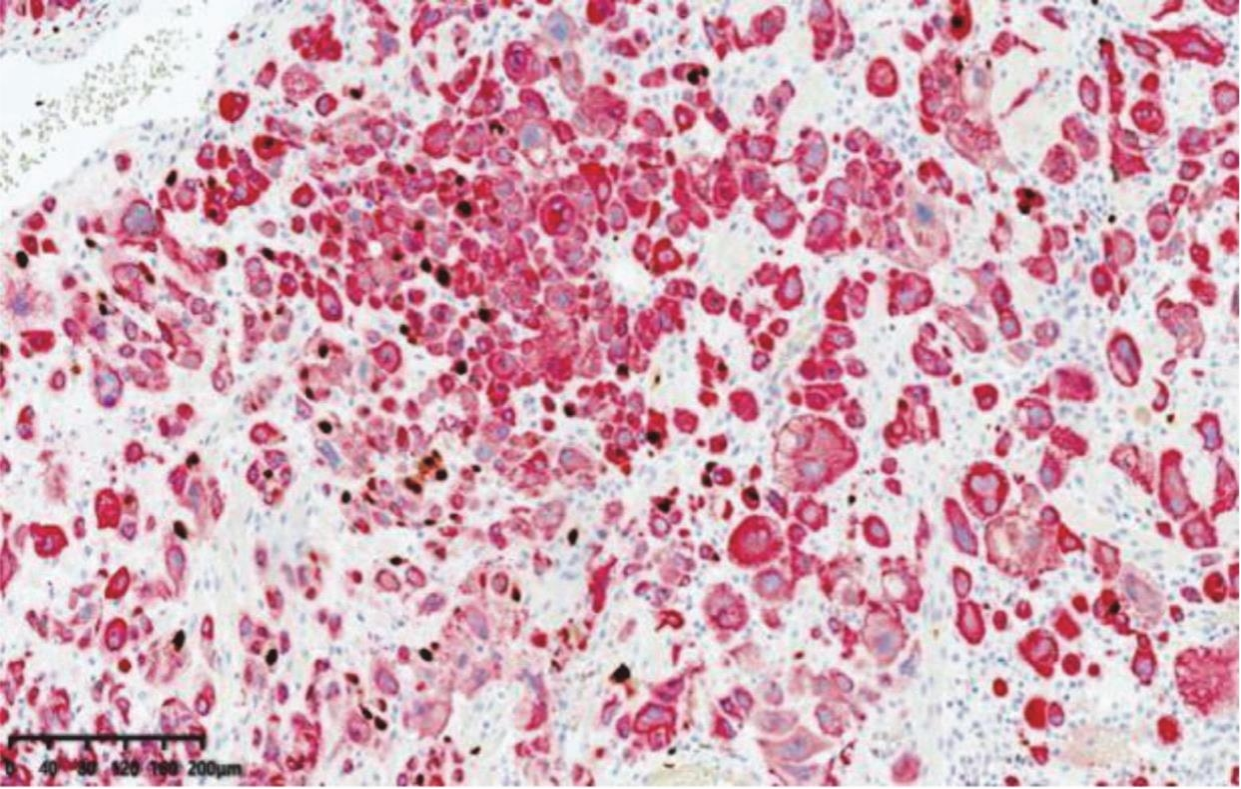
(b)

## 3. Discussion

This article reports two cases of PSTT. The patients exhibited significant differences in their main symptoms, the time interval from the last delivery to disease onset, the degree of serum HCG elevation, and the presence of vaginal metastasis. The diagnosis and treatment process were complex. However, after pathological confirmation, both patients achieved good therapeutic outcomes through surgery and adjuvant chemotherapy. PSTT is rare, with highly heterogeneous clinical manifestations and challenging diagnosis and treatment. Therefore, we have summarized the diagnosis and treatment process of these patients and conducted a literature review.

PSTT is the rarest form of GTN, accounting for 2.8%–3.0% of GTN cases, with an incidence rate of ~0.01–0.30 per 100,000 pregnancies. It was first described by Kurman in 1976 and officially named PSTT in 1981. Its biological behavior is diverse, and most current literature reports are case studies, with a total of over 300 cases reported [1, 2]. The average age at diagnosis for patients is 29–35 years, with a median interval of 3–36 months from the last pregnancy. Approximately 61.5% of cases occur after full‐term pregnancies. In this report, the patients were aged 28 and 31 years, respectively, and both had full‐term cesarean deliveries, with intervals of 1 month and 3 years, respectively, which is consistent with the literature [3]. The most common symptom of PSTT is vaginal bleeding (Case 1), followed by amenorrhea (Case 2). The main physical sign is uterine enlargement. Additionally, ~10% of cases are associated with nephrotic syndrome [4], which may be due to cytokines released by tumor cells that activate the coagulation system and form immune complexes, leading to their deposition in the glomerular filtration membrane.

The clinical characteristics of PSTT are markedly heterogeneous, encompassing significant variations in clinical presentation, imaging features, prognosis, and treatment response. This heterogeneity limits the accuracy of conventional noninvasive diagnostic modalities (such as serum tumor markers and ultrasonography) [5], underscoring the indispensable role of histopathological examination as the diagnostic gold standard. Based on a comprehensive literature review, diagnostic approaches for PSTT are summarized as follows:

• Serum HCG: ~80% of PSTT patients exhibit mild to moderate elevations in serum HCG (ranging from 5 to 26,000 mIU/mL). In the two cases reported here, baseline serum HCG levels fluctuated between 8000 and 14,000 mIU/mL and 30–40 mIU/mL, respectively. This contrasts with the typically high HCG levels seen in choriocarcinoma [6]. Serum HCG levels do not correlate with tumor burden or malignancy but can be used to assess treatment efficacy during follow‐up.
• Imaging Studies:
 Ultrasound: Color Doppler ultrasound is the first‐line imaging modality, assessing the location, size, cystic or solid nature, blood flow, and myometrial invasion of the lesion. PSTT ultrasound findings can be categorized into three types: Type I shows an inhomogeneous solid mass within the uterine cavity with mild to moderate vascularization; Type II reveals an inhomogeneous solid mass within the myometrium with variable vascularization; Type III features cystic lesions within the myometrium with high vascularization (cavitary lesions) [7].
 MRI: Pelvic MRI provides more precise localization of uterine lesions and myometrial invasion depth compared to ultrasound. It can also effectively evaluate pelvic soft tissue metastasis and pelvic lymph node involvement.
 CT: Abdominal and pelvic CT, along with pulmonary CT, are used for clinical staging, assessing metastatic lesions, and detecting disease recurrence. The use of PET‐CT is controversial and generally reserved for patients with distant metastasis or recurrence [8].
• Pathology and Immunohistochemistry: Unlike invasive mole and choriocarcinoma, PSTT diagnosis primarily relies on histopathology and immunohistochemistry. It is important to note that a negative diagnostic curettage does not completely rule out PSTT. Data from Peking Union Medical College Hospital indicate that the positive rate of diagnostic curettage in patients who were eventually diagnosed with PSTT after hysterectomy is only 40% [9]. Therefore, for patients highly suspected of having PSTT, close monitoring of serum HCG and imaging studies is necessary after diagnostic curettage. Further lesion resection or hysterectomy may be required to confirm the diagnosis if needed.

Imaging modalities are prone to misdiagnosis or missed diagnosis of PSTT, while HCG, as a tumor marker, cannot reliably differentiate PSTT from other GTN subtypes. Histopathology, through analysis of trophoblastic morphology, arrangement, and depth of infiltration, enables differentiation of PSTT from epithelioid trophoblastic tumor (ETT) and atypical placental site nodule (APSN). This distinction is crucial, as prognosis and therapeutic strategies differ significantly among these subtypes [10]. Grossly, PSTT typically presents as a nodular, round, solid mass. The cut surface is usually solid and fleshy, ranging in color from white to pale yellow. Deep myometrial invasion and local hemorrhage and necrosis are observed in 50% of cases, but involvement of the broad ligament and adnexa is rare [5]. Microscopically, intermediate trophoblasts are visible at the implantation site. These are large, polyhedral to round, usually mononuclear cells. In more than two‐thirds of cases, tumor cells replace the walls of myometrial blood vessels. This vascular transformation is unique among all human solid tumors [6]. In Case 2 of this report, typical thick‐walled vessels replaced by trophoblasts were observed (Figure 9), which is a characteristic feature of PSTT.

Immunohistochemically, PSTT usually shows diffuse positivity for hPL and CK, and positivity for CD146, CD10, and human leukocyte antigen G (HLA‐G). HCG and placental alkaline phosphatase (PLAP) are only focally positive or weakly positive. CD117, P63, and SALL4 are negative. The Ki‐67 proliferation index is typically 10%–30%. In contrast, placental site exaggerated reaction, a benign tumor‐like lesion with the same origin as PSTT, usually shows a negative Ki‐67, while choriocarcinoma often has a Ki‐67 proliferation index greater than 50% [9]. The latest edition of the WHO guidelines indicates that dual staining for CD146 and Ki‐67 is the most useful immunohistochemical method for diagnosing PSTT [3, 11, 12]. Both cases in this report exhibited the characteristic immunohistochemical features of PSTT. The low Ki‐67 proliferation index of only 5% in Case 2 may be related to pretreatment with mifepristone.

PSTT exhibits heterogeneous biological behavior, with some cases demonstrating high aggressiveness while others follow an indolent course. Furthermore, individual patient needs, such as fertility preservation, add complexity to therapeutic decision‐making [13]. For early‐stage PSTT, total hysterectomy is the preferred treatment option because PSTT is relatively insensitive to chemotherapy compared to other forms of GTN. For young women who wish to preserve their fertility, fertility‐sparing approaches should only be considered in carefully selected patients after thorough and extensive counseling, as patients with diffuse disease are not ideal candidates for conservative surgical treatment [1]. Previous studies and the National Comprehensive Cancer Network (NCCN) guidelines suggest that for Stage I PSTT patients who have undergone total hysterectomy, adjuvant chemotherapy is recommended if any of the following factors are present: deep myometrial invasion of the tumor, extensive hemorrhage and necrosis of the tumor, or a tumor nuclear mitotic count > 5/10 HPF [14, 15]. Given the poor sensitivity of PSTT to chemotherapy, combination chemotherapy regimens should be used. Commonly used chemotherapy regimens include 5‐fluorouracil (5FU) + actinomycin D (KSM) and EMA‐CO. For patients with poor response or resistance to these regimens, the EMA‐EP regimen can be considered [16, 17]. In this report, both patients had completed childbearing and had tumors that invaded the deep myometrium; therefore, they underwent hysterectomy followed by adjuvant chemotherapy with the EMA‐CO regimen, achieving good therapeutic outcomes.

For patients with Stage II–IV disease and metastatic lesions, combined chemotherapy is generally recommended after hysterectomy, with metastasectomy as needed [3]. In recent years, targeted therapies with inhibitors of programmed death‐ligand 1 (PD‐L1) and its receptor PD‐1 have emerged as a new treatment strategy for refractory or resistant PSTT, suggests that in patients with pathologically confirmed early‐stage PSTT and PD‐L1 positivity on immunohistochemistry, immunotherapy may represent an alternative to hysterectomy. However, these have been reported only in case studies [16, 18] and require further investigation regarding the rarity and heterogeneity of PSTT.

## 4. Conclusion

PSTT is rare and lacks specific clinical manifestations, which can easily lead to misdiagnosis or missed diagnosis. In any woman of childbearing age presenting with abnormal vaginal bleeding and mild to moderate elevation of serum HCG, the possibility of PSTT should be considered. Ultrasound and pelvic MRI are important, while the definitive diagnosis relies on pathology and immunohistochemistry. Key diagnostic criteria include the replacement of vascular walls by trophoblastic cells, positivity for CD146, and a Ki‐67 proliferation index of 10%–0%. When the disease is confined to the uterus, hysterectomy is the primary treatment method. For patients with high‐risk factors, adjuvant multiagent chemotherapy is recommended after surgery.

## Author Contributions

**Yiqi Guan:** writing – original draft, formal analysis. **Jinsong Han:** writing – methodology, revision. **Kun Zhang and Yiting Wang:** supervision.

## Acknowledgments

No AI software have been used to prepare the manuscript.

## Funding

The authors received no funding from an external source.

## Disclosure

All authors declare that we agree to the publication of the research findings presented in this manuscript in Case Reports in Obstetrics and Gynecology. The authors have carefully read and understood the submission guidelines and requirements of the journal. They certify that the data, figures, and images presented in this manuscript are original.

## Conflicts of Interest

The authors declare no conflicts of interest.

## Data Availability

The data that support the findings of this study are available from the corresponding author upon reasonable request.
